# Dr. Theodore H. Boysen

**Published:** 1903-06

**Authors:** 


### OBITUARY.

Dr. Theodore H. Boysen, of Egg Harbor City, N. J., a graduate of the University of Buffalo, class of 1874, died at his home March 12, 1903, of chronic valvular disease of the heart, aged 49 years. He was born in Rogersville, O., January 14, 1854, and was the son of Dr. Otto Boysen, who afterward removed to Buffalo and engaged in the practice of medicine. After graduation, Dr. Theodore Boysen began the practice of his profession in this city, remaining for two years, when he removed to Egg Harbor City. Dr. Boysen became a leading practitioner in his locality, where he acquired a large clientèle and commanded the universal respect of the community. He is survived by a son, Dr. Theodore H. Boysen, Jr., who will assume his father's practice.

